# Pediatric Glomangioma of the Scalp

**DOI:** 10.7759/cureus.93218

**Published:** 2025-09-25

**Authors:** Hugues Bulteau, Mélodie-Anne Karnoub

**Affiliations:** 1 Neurosurgery, Roger Salengro Hospital - Centre Hospitalier Universitaire de Lille (CHU Lille), Lille, FRA; 2 Pediatric Neurosurgery, Roger Salengro Hospital - Centre Hospitalier Universitaire de Lille (CHU Lille), Lille, FRA

**Keywords:** cranial vault tumor, glomangioma, glomus tumor, pediatric glomangioma, pediatric neurosurgery

## Abstract

We report the case of a 14-year-old boy who has been followed since four years ago for an extra-osseous cranial vault tumor, which has been removed due to its increase in size and its painful nature. The outcomes were marked by regression of the pain and the absence of recurrence. We described the clinical signs, MRI characteristics, surgery findings, and anatomopathological descriptions of this pediatric glomangioma and carried out a literature review about all the glomus tumors and cranial glomus tumors. It is the only case reported in the current literature on this topic.

## Introduction

A glomus tumor (or chemodectomas or non-chromatograffin paragangliomas) is a neoplasm that arises from the glomus body [1], a thermoregulatory neurovascular entity. This arteriovenous structure is implicated in temperature regulation by altering blood flow to the skin [1].

The glomus body is composed of glomus cells, capillaries, and smooth muscle. Consequently, there are three subtypes of glomus body tumors: solid glomus tumor, glomangioma, and glomangiomyoma [1].

## Case presentation

A 14-year-old boy who has been followed since 2018 for a cranial vault tumor, next to the parietal bone but extra-osseous, only in the scalp, without intracranial extension. Clinically, the tumor was reluctant and sensitive and initially considered a scalp capillary angioma. Its size was increasing; moreover, the tumor became painful, which is why we decided to remove it.

**Figure 1:**
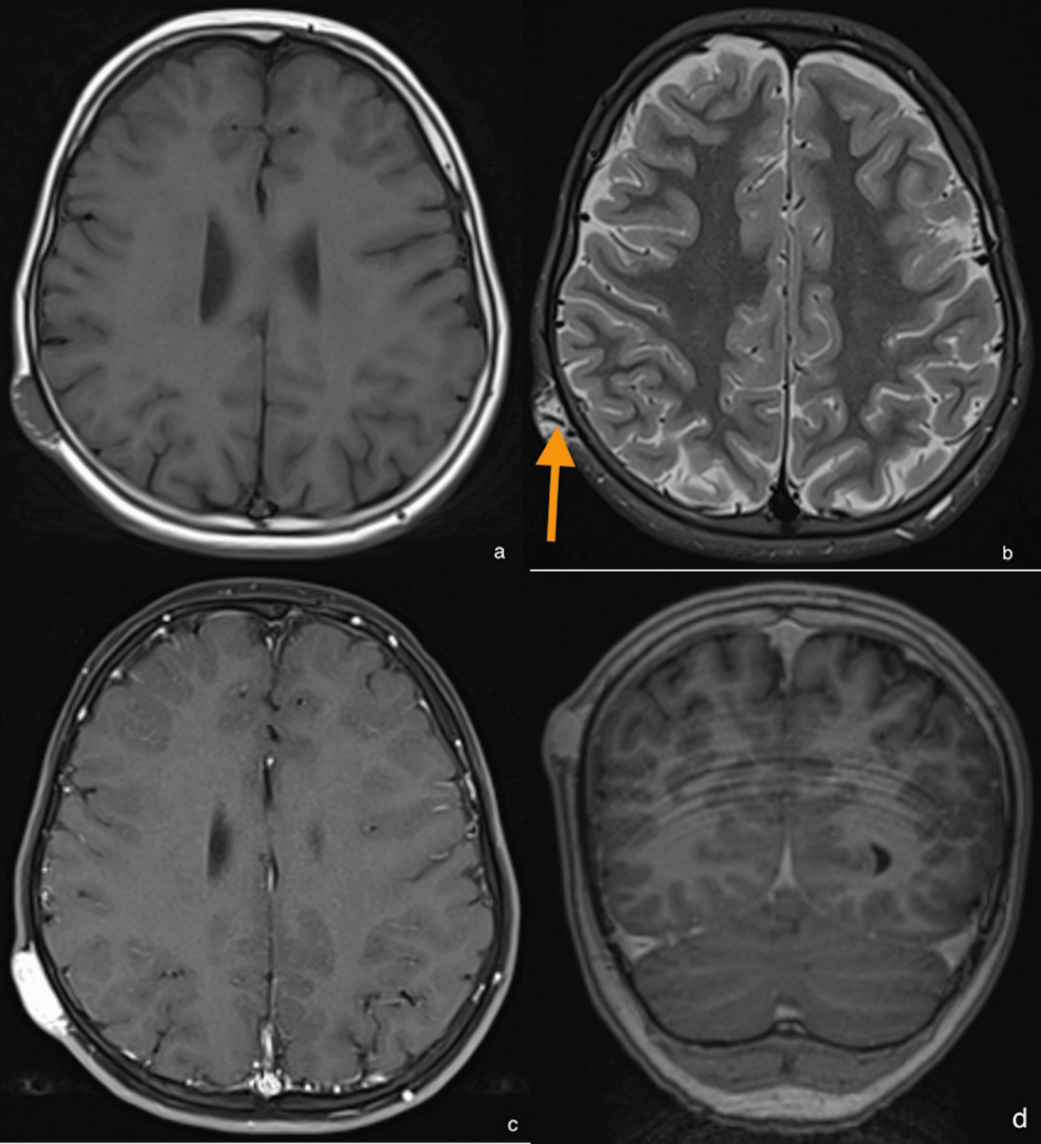
MRI caracteristics (a) Axial T1 hyposignal; (b) axial T2 STIR shows a big intratumoral vessel; (c) and (d) axial and coronal T1 gadolinium hypersignal

An MRI was performed and showed (Figure 1) a hypoT1 and hyperT2 tumor, with intense, early homogeneous and durable enhancement with gadolinium, hypervascular; moreover, we saw flow voids, confirming that the lesion did not invade the bone vault and the absence of an intracranial lesion.

**Figure 2:**
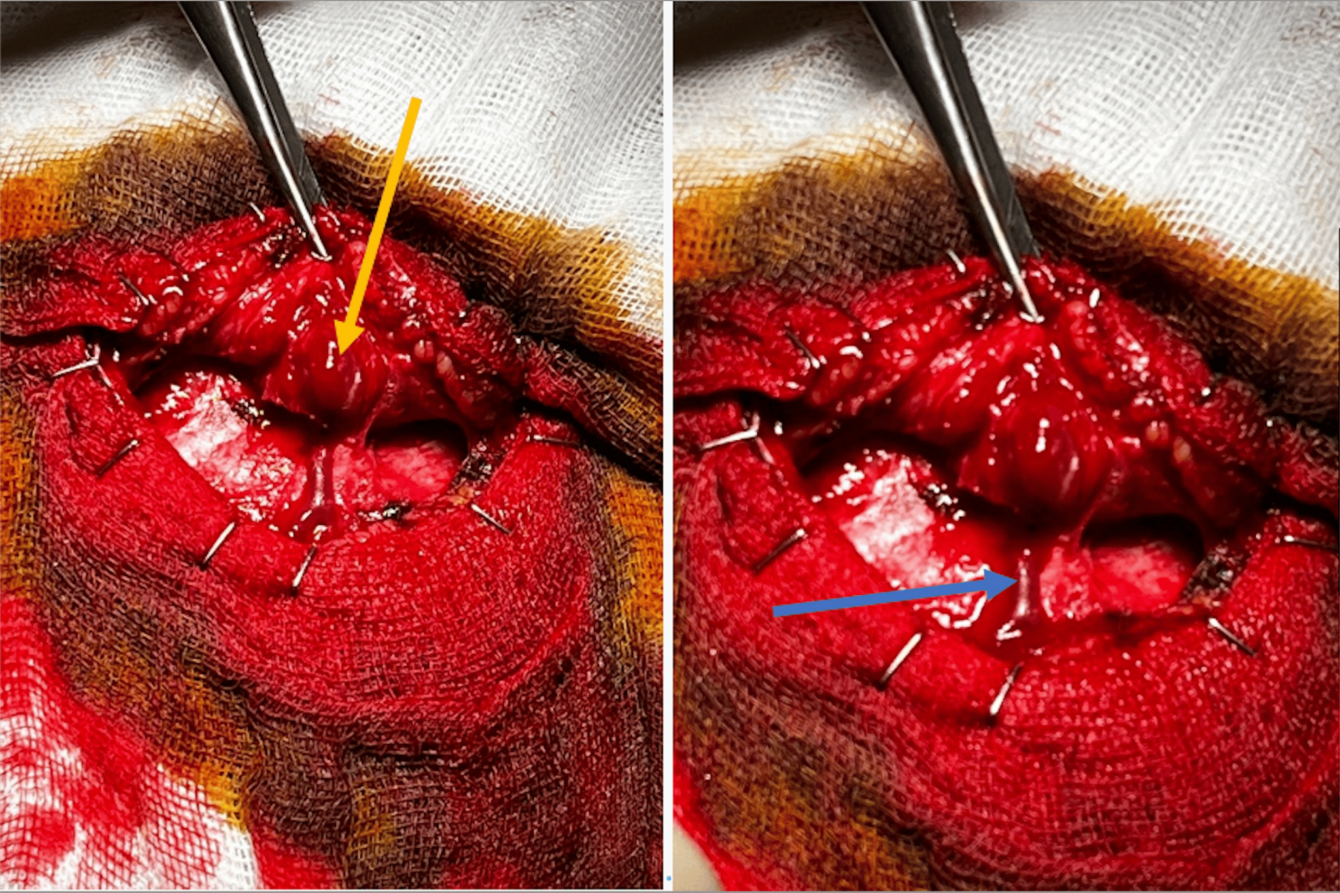
Surgical considerations These two images illustrate the nidus (yellow arrow) and a prominent venous drainage (blue arrow).

The surgery was not complicated (Figure 2) and was performed via a curvilinear incision centered on the lesion, coagulation of the arterial afference, and a large venous efference, resection of the nidus; the tumor was hemorrhagic.

The post-operative course was uneventful. The follow-up takes place with a clinical control at two months, clinical control plus MRI at six months, and clinical control at three years; both of them were satisfying, without any clinical symptoms or remnographic recurrence.

**Figure 3:**
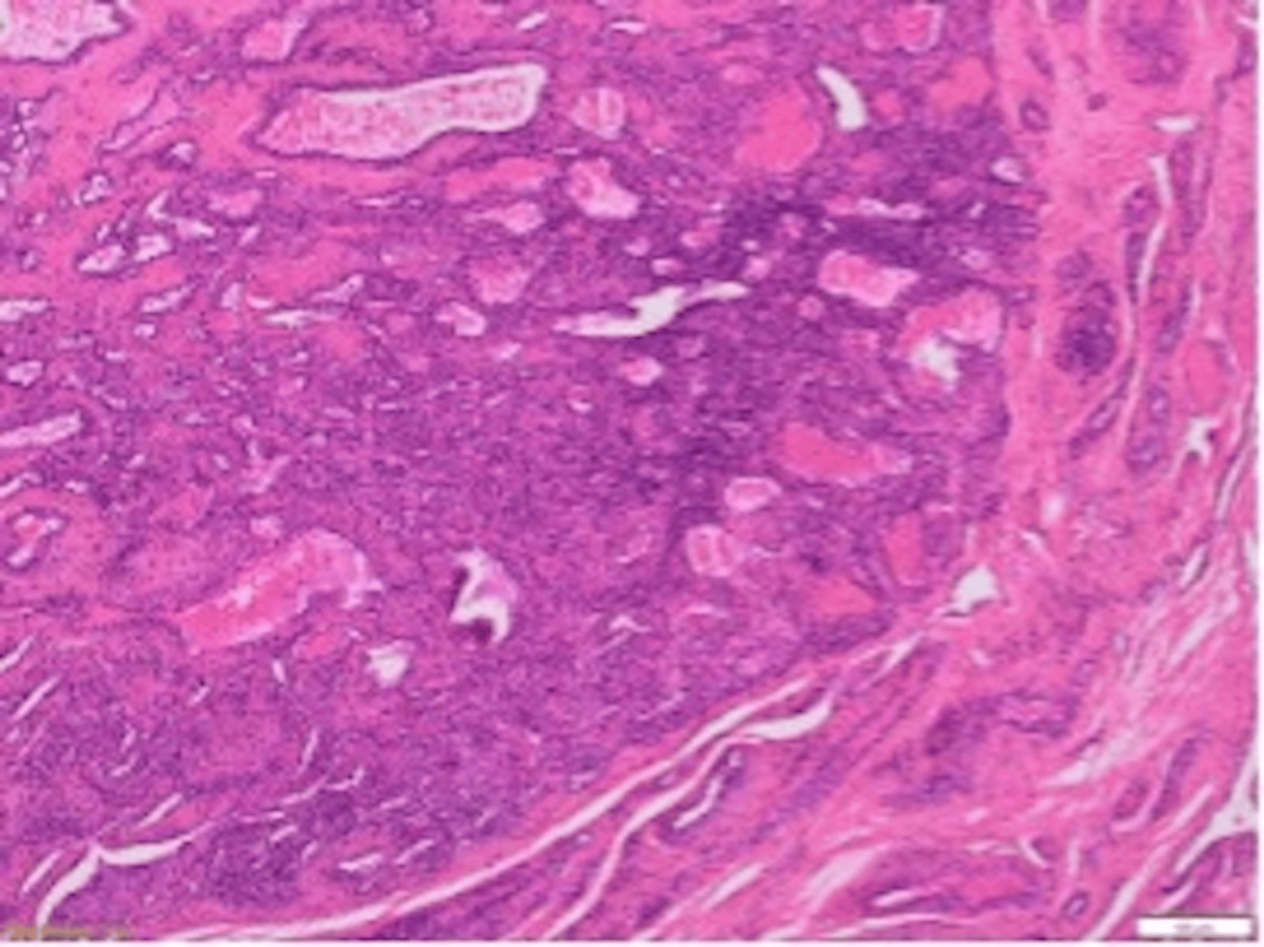
Histological appearance after hematoxylin-eosin-safranin (HES) staining Magnification: x50

**Figure 4:**
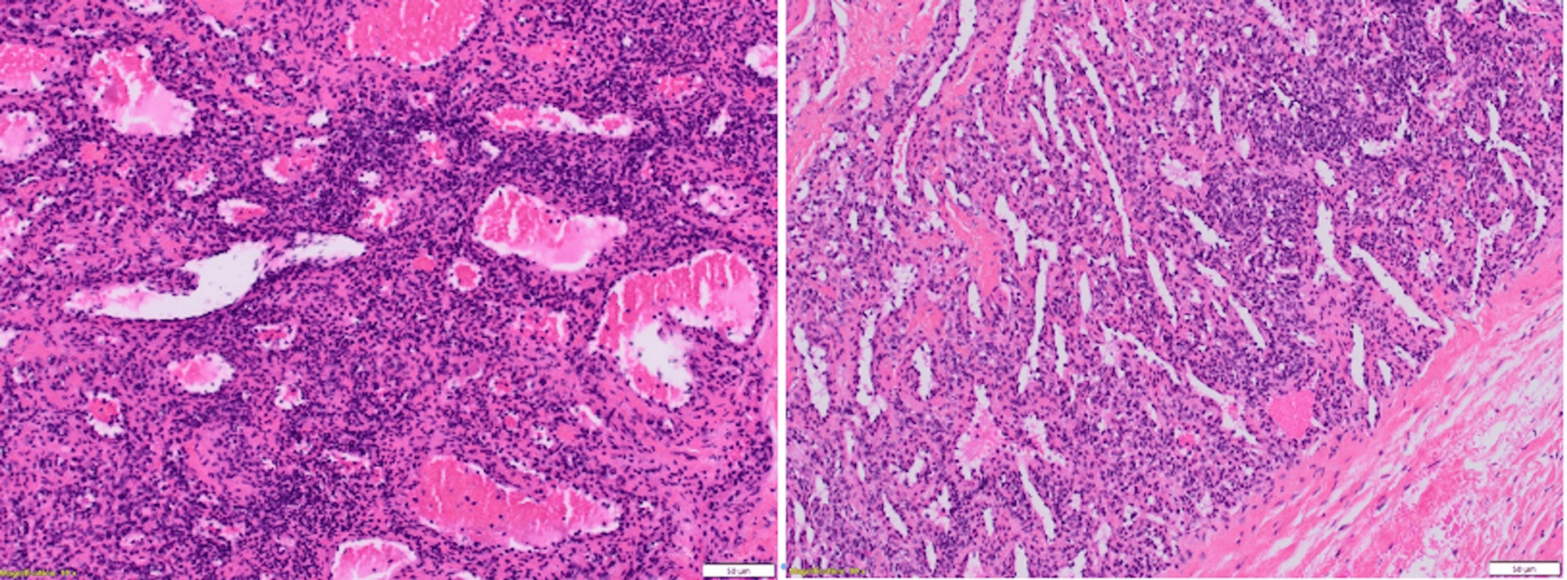
Histological appearance after hematoxylin-eosin-safranin (HES) staining Magnification: x100

**Figure 5:**
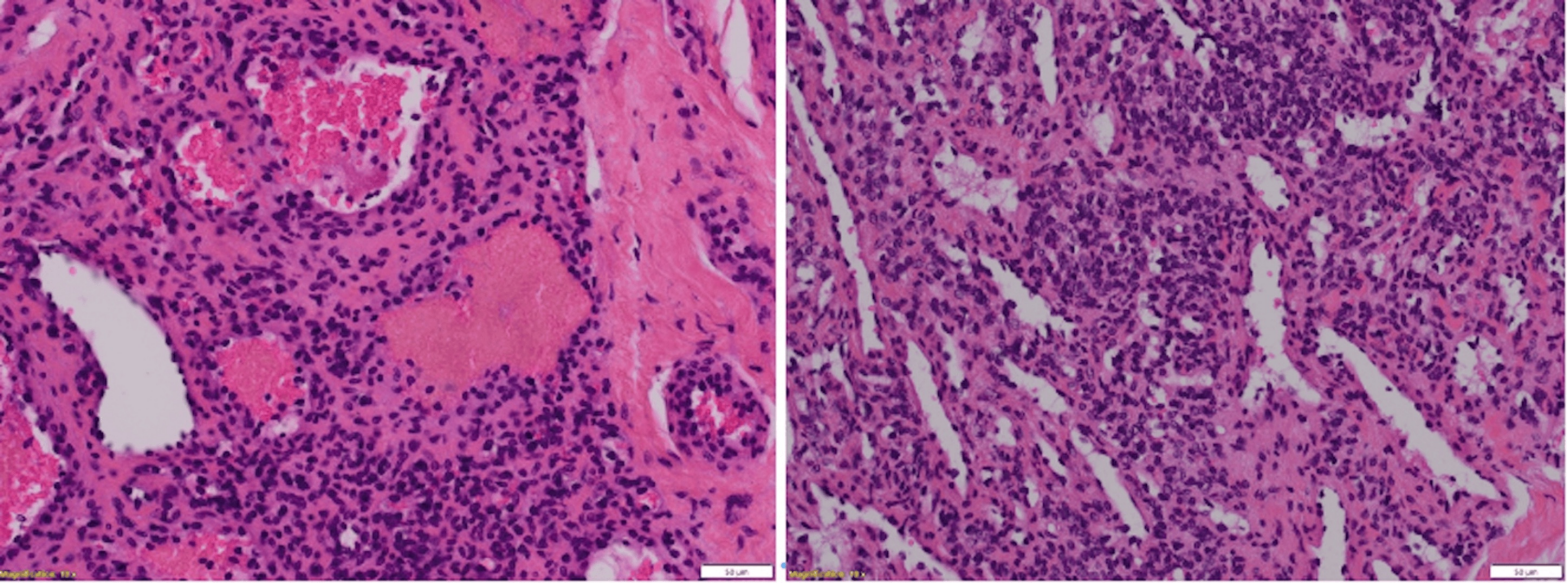
Histological appearance after hematoxylin-eosin-safranin (HES) staining Magnification: x200 Tumor proliferation comprising numerous veins of variable caliber comprising a cell population in their wall Population of spindle or rounded cells lacking atypia

**Figure 6:**
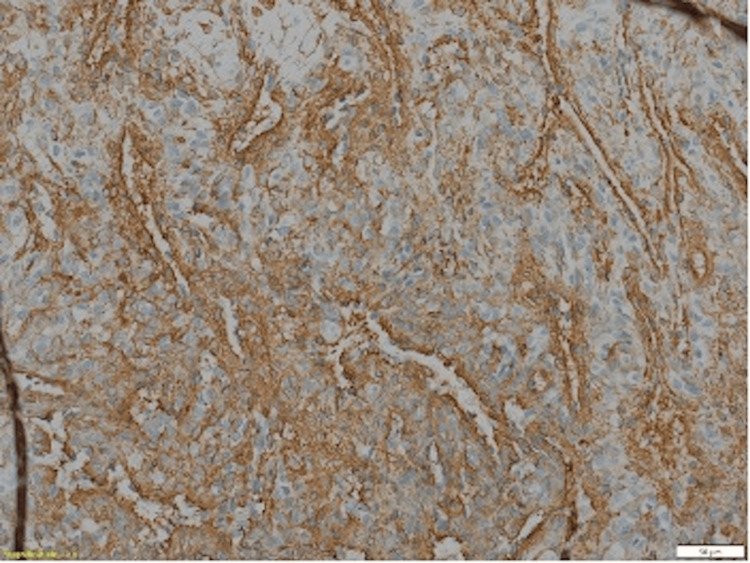
Cells marking Actine muscle lisse + : marking of the spindle-shaped or rounded cells in the vessel wall

**Figure 7:**
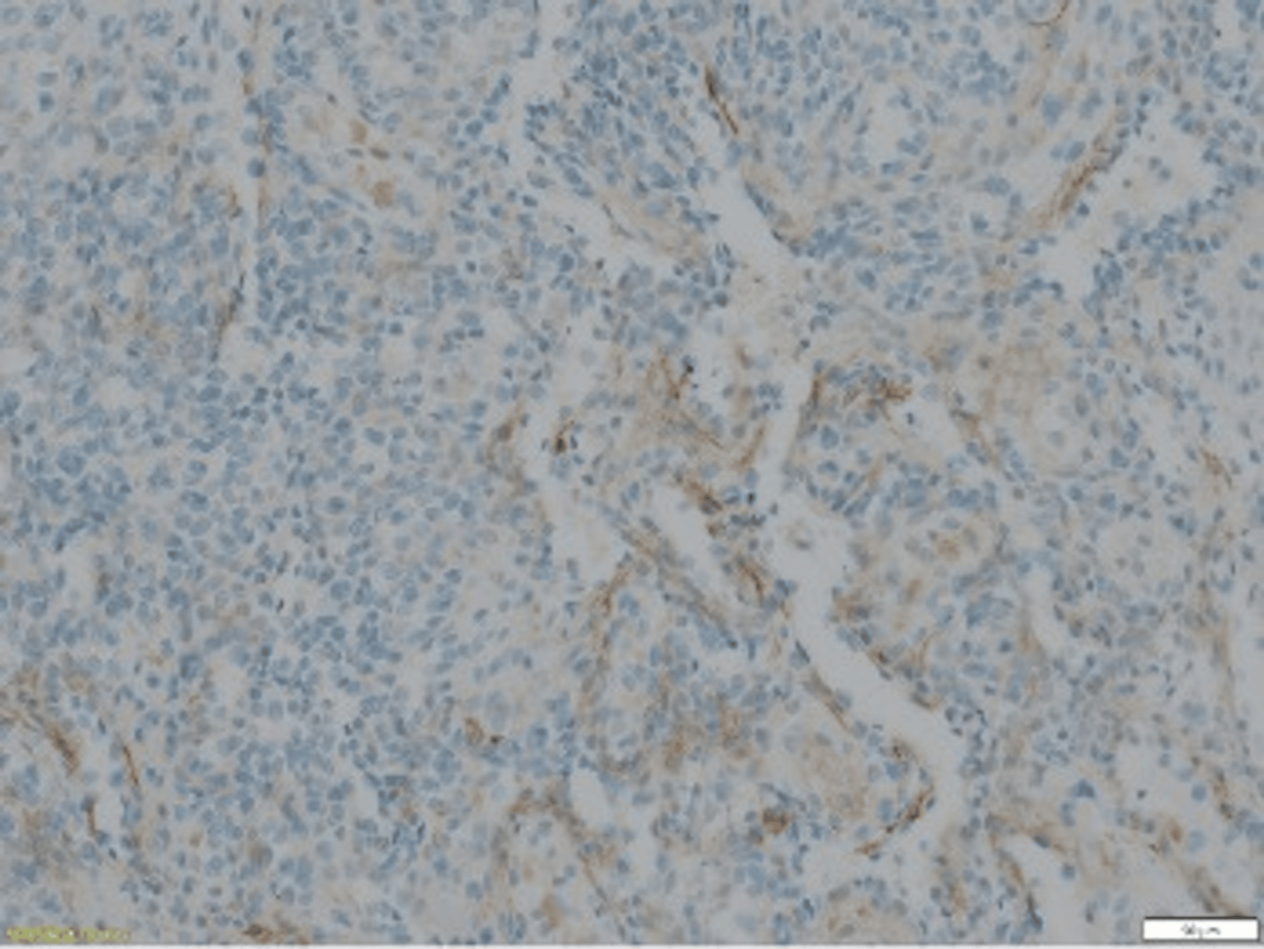
Cells marking Caldesmone - : absence of marking of spindle or rounded cells in the vessel wall (wall marked by caldesmone)

**Figure 8:**
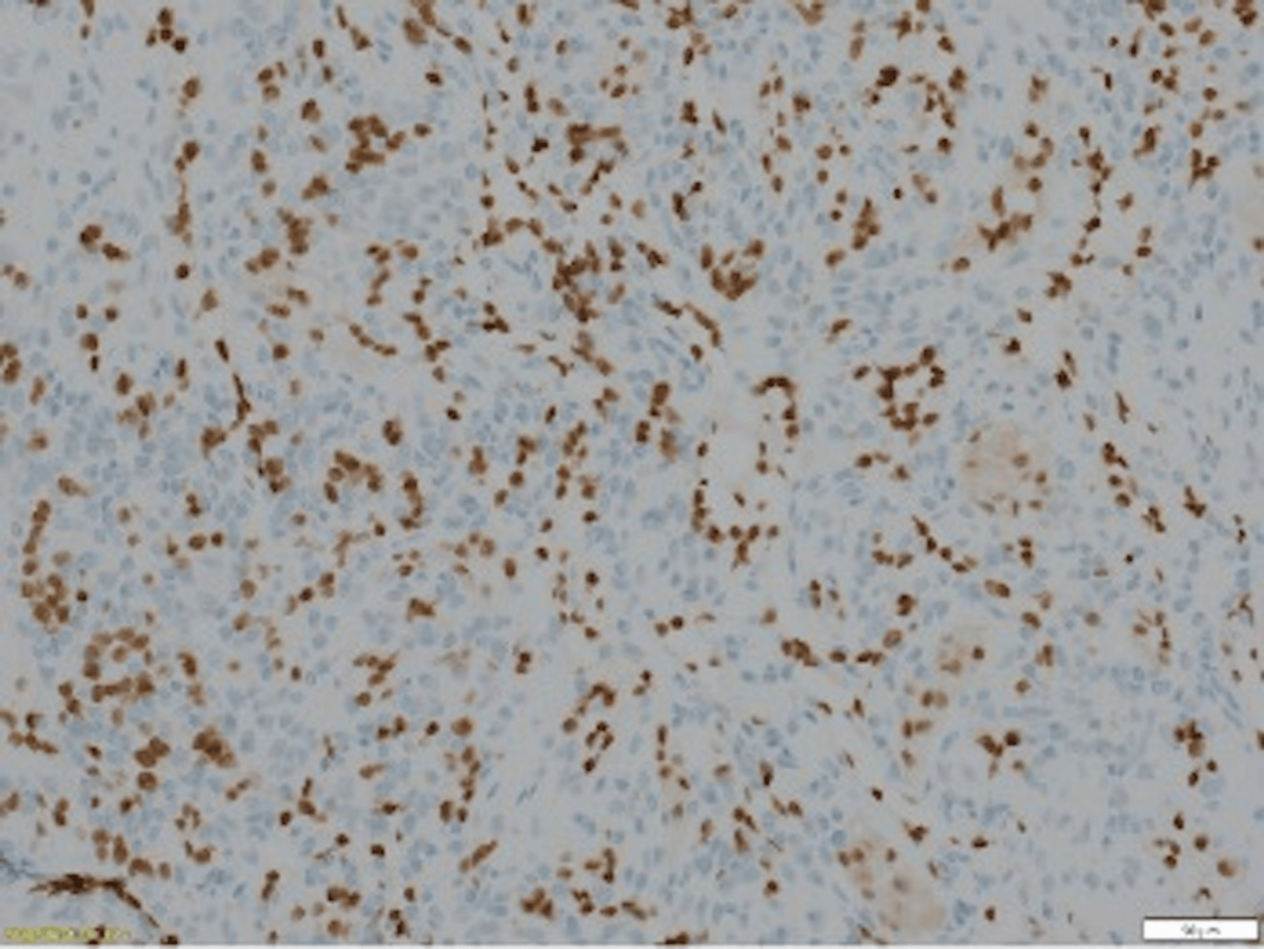
Cells marking ERG - : marking of the vessel wall without marking of spinded or rounded cells

The anatomopathology (Figures 3-5) analysis came back in favor of a benign tumor without signs of malignancy, and the immunohistochemical analysis shows tumoral cells diffusely marked by anti-smooth muscle actin antibody and weak heterogeneous labeling by the anti-caldesmon antibody, which gave us the diagnosis (Figures 6-8).

## Discussion

Literature review

About All the Glomus Tumors

The glomus tumors are most of the time localized on the digits, but they can sometimes appear in other locations [1]. The most common time of occurrence is in childhood and adolescence. There are sometimes familial forms with mutations in glomulin genes. The symptoms are composed of a classic triad: pain, cold sensitivity, and tenderness. They can rarely undergo malignant degeneration, and a few cases of malignant glomus tumor with regional or distant dissemination have been reported. They represent an issue because there is often a long time before diagnosis, which is why they have to be known.

About Cranial Glomus Tumors

They can be confused with the head and neck paragangliomas, glomangiopericytoma, and sinonasal-type hemangiopericytoma. Four locations of glomus development are particularly common: (1) the inferior ganglion region (ganglion nodosum) and cervical portion of the vagus nerve (glomus vagale or vagal body tumor); (2) the carotid bifurcation (carotid body tumor); (3) the middle ear cavity (glomus tympanicum tumor); and (4) the jugular bulb region (glomus jugulare tumor) [2].

In the head and neck regions, glomus tumors commonly exert compressive effects on adjacent blood vessels and cranial nerves, which is why radiosurgery is sometimes proposed as a treatment. Conventional radiotherapy is effective too, but with this treatment, adjacent cranial nerves and vessels and critically neurologic structures are exposed to radiation doses similar to those received by the tumor. There are between 0 and 15% complications with radiosurgery according to Lee et al. in radiosurgery for glomus tumors [2].

There are very few cases of progression, and, even if the efficacy seems to be obvious, no relationship is observed between radiation dose and long-term tumor control in a multicenter series published by the International Gamma Knife Radiosurgery Foundation in 2012 [2,3]. The primary limitation of radiosurgery in treating glomus tumors is related to tumor location [2]. Radiosurgery GammaKnife is not ideally suited for extensive extracranial glomus tumors (e.g., vagal body tumors and carotid body tumors) due to patient immobilization constraints. In these situations, when open neck dissection is contraindicated, linear accelerator-based radiosurgery (e.g., Cyberknife) or fractionated radiotherapy constitute viable alternatives.

Craniotomy for tissue biopsy is seldom required in cases that involve a glomus jugulare or tympanicum tumor, given their characteristic radiological appearance and typical anatomical localization of the neoplasm, which are usually sufficient for diagnosis.

Some Other Sites Along the Neuraxis

Asa et al. described a case of a sellar glomangioma [4]. The hypothesis was that the gomitoli of the hypophyseal portal vessels are structures that resemble glomera found in other sites, and derivation of this tumor from gomitoli is suggested [4]. Gresham et al. [5] reported a case of a paranasal sinus glomangioma (an ethmoid sinus glomangioma with intracranial extension) that induced osteomalacia by paraneoplastic phenomenon, whose symptoms have been completely resolved with surgery.

Additionally, a case of a paravertebral glomangioma, initially misdiagnosed as a schwannoma, has been described by Babeau et al. [6]. These tumors are usually well delimited, although osteolysis and epidural extension can sometimes be found.

## Conclusions

Glomangioma is a rare and often underrecognized vascular tumor of the cranial region. This rare tumor needs to be known, as well as its typical clinical presentation and its MRI appearance, especially before surgery, since it is a hemorrhagic tumor. Further molecular and pathological characterization could also provide new insights into the pathogenesis of pediatric cranial glomangiomas, potentially guiding future therapeutic approaches.

It remains a little-known entity, and we hope that more studies and cases will be reported in the literature in order to limit frequent diagnostic wandering in this type of tumor, which can be simple to treat. Multidisciplinary collaboration, particularly between pediatricians, radiologists, and neurosurgeons, is essential to ensure timely diagnosis and optimal management of these rare lesions. Raising awareness among clinicians is crucial, as early recognition may prevent unnecessary investigations, facilitate appropriate surgical planning, and ultimately improve patient outcomes.
